# Case report: Endovascular intervention of internal carotid artery pseudoaneurysm secondary to nasopharyngeal carcinoma radiotherapy

**DOI:** 10.3389/fsurg.2022.1099416

**Published:** 2023-01-06

**Authors:** Chao Li, Jiachao Lu, Yu Luo, Daqin Feng

**Affiliations:** Department of Neurosurgery, The First Affiliated Hospital, Guangxi Medical University, Nanning, China

**Keywords:** internal carotid artery pseudoaneurysm, nasopharyngeal carcinoma, nasal bleeding, endovascular intervention, case report

## Abstract

**Background:**

Internal carotid artery pseudoaneurysm (PSA) is a serious complication after radiotherapy for nasopharyngeal carcinoma, and once it ruptures and bleeds, it will seriously affect the patient's survival and prognosis. However, because of its relatively low incidence, many medical institutions lack experience in managing this type of emergency.

**Case information:**

In this case report, we described two cases suffered ruptured internal carotid artery PSA after radiotherapy for nasopharyngeal carcinoma, including their history, diagnosis, and treatment. Both cases underwent emergency endovascular interventions, one of which with long-term healing after embolization of the PSA, and the other one with re-bleeding after embolization and was eventually stopped by embolization of the parent artery. Ultimately, both cases received timely and effective treatment.

**Conclusion:**

This case report detailed the diagnosis and treatment course of internal carotid artery PSA after radiotherapy for nasopharyngeal carcinoma, which enhanced the understanding of this emergency, and provided valuable information and experience for the treatment strategy of similar PSA on the internal carotid artery.

## Introduction

Nasopharyngeal carcinoma is a common malignant tumor in southern China, because of its complex anatomy and high sensitivity to radiotherapy, the current clinical treatment adopts a comprehensive treatment based on radiotherapy, with a 5-year survival rate of 50% to 80% (1–3). With the prolongation of patient survival, nasopharyngeal haemorrhage caused after radiotherapy becomes a serious complication affecting the prognosis of patients. The incidence of nasopharyngeal hemorrhage after radiotherapy has been reported to be 2.7%, and ruptured internal carotid artery pseudoaneurysm bleeding accounts for approximately 1% of patients with hemorrhage (4, 5). The hemorrhage is characterized by rapid, recurrent, massive and not easily controlled, and if resuscitation is not timely, the patient may die within a very short time due to asphyxia or circulatory collapse (6). However, for bleeding from pseudoaneurysms of the internal carotid artery, relying on simple nasal tamponade or surgical ligation often fails to solve the problem, and endovascular intervention is now a more effective treatment for this type of bleeding (7, 8). We will show two typical cases of endovascular intervention for bleeding from pseudoaneurysm secondary to nasopharyngeal carcinoma radiotherapy. This case report follows the CARE Guidelines (9).

## Case presentation

### Case one

**Figure 1:**
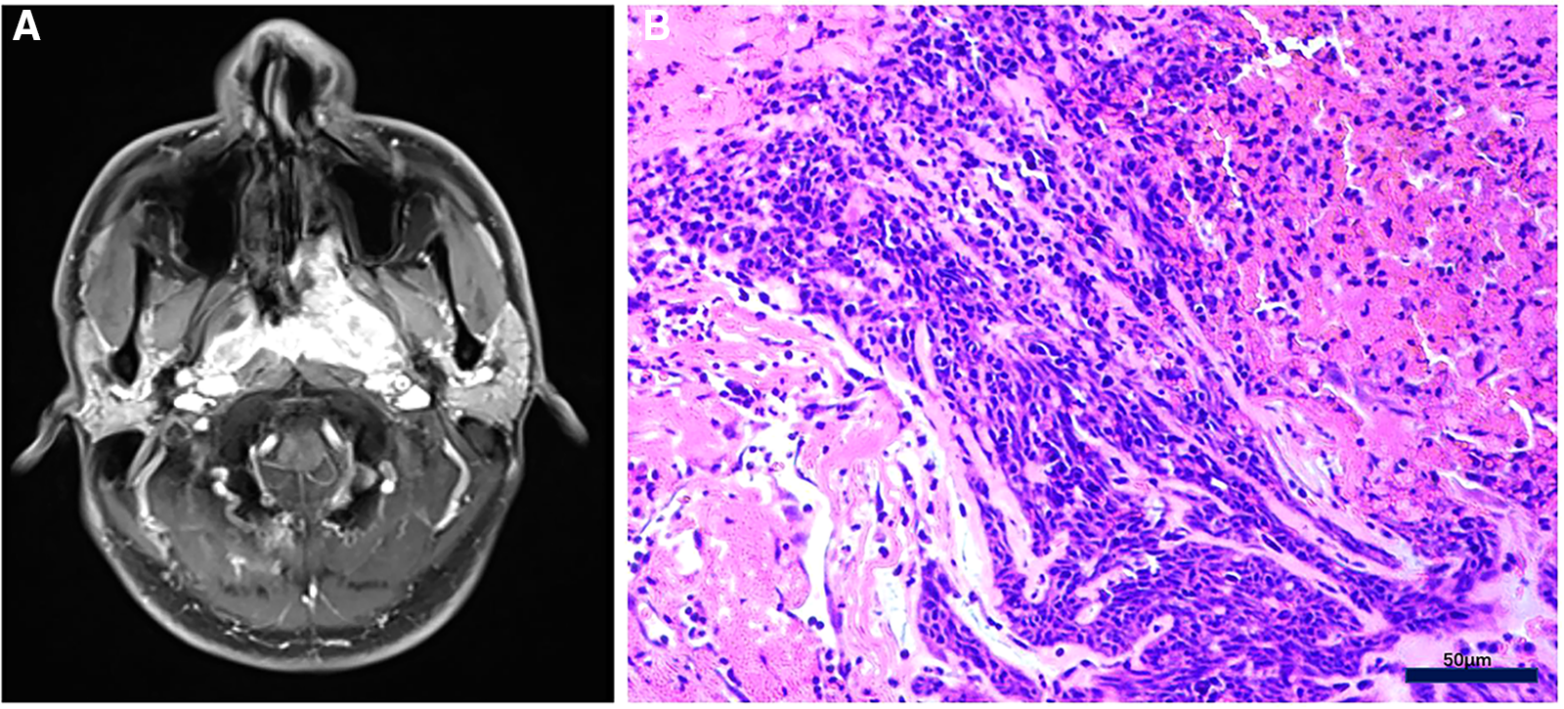
(**A**) MRI showed that nasopharyngeal carcinoma invaded the left postnaris, pterygopalatine fossa, cavernous sinus and skull base. (**B**) Morphological observation by HE staining was consistent with the diagnosis of undifferentiated non-keratinizing nasopharyngeal carcinoma, with no Keratinized beads of squamous cell carcinoma positive staining. Bar, 50 *μ*m.

**Figure 2:**
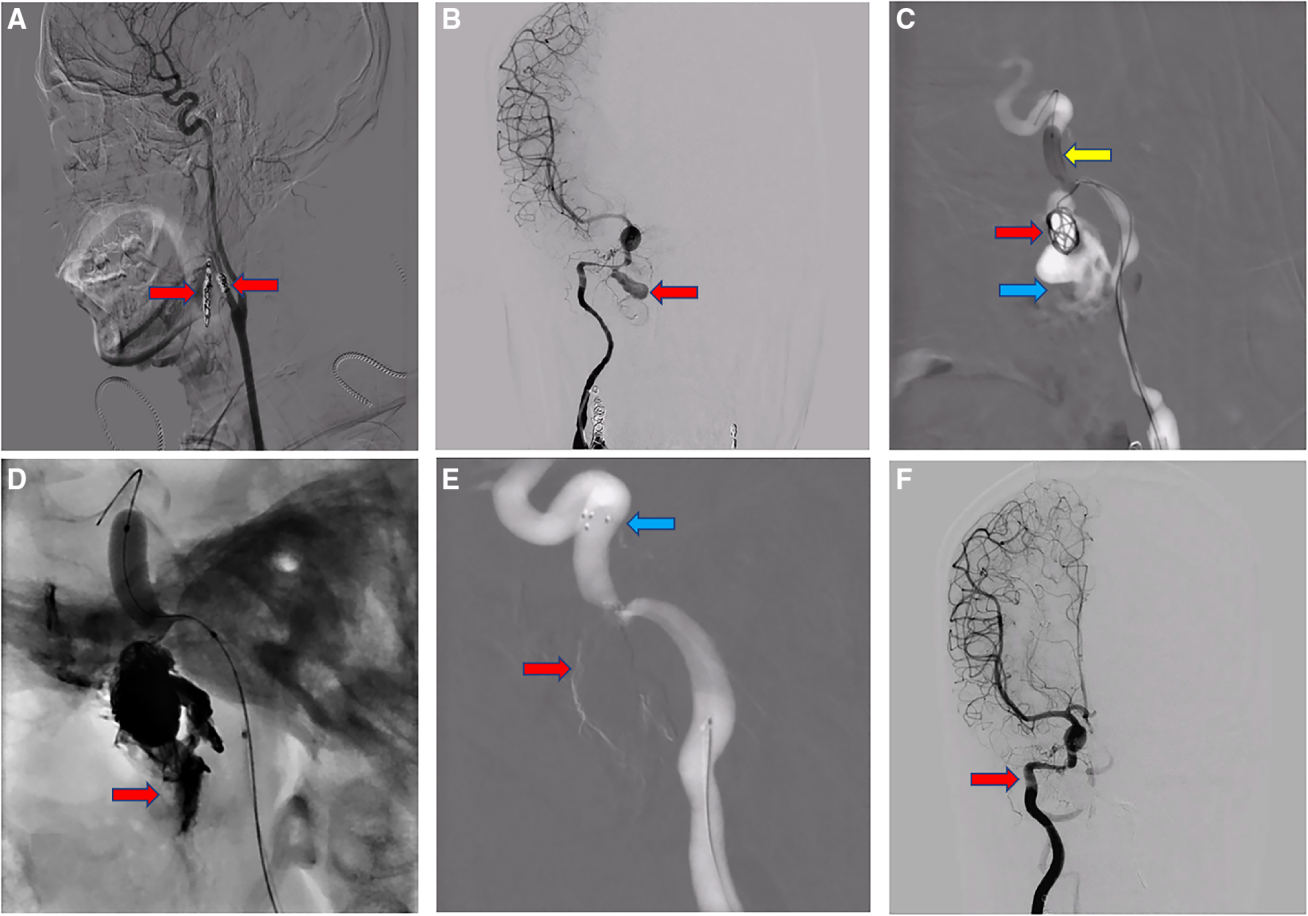
(**A**) bilateral external carotid arteries embolization with spring coil (red arrow). (**B**) Angiography suggests a narrow neck pseudoaneurysm in the right internal carotid artery rupture hole segment, diameter about 9.6 X 15.7 mm (red arrow). (**C**) Extravasation (blue arrow) of contrast medium into the nasal cavity was seen at the base, lateral and neck part of the aneurysm; The microcatheter was delivered into the aneurysm cavity, and the balloon (yellow arrow) was guided to the C4 segment and opened with a certain pressure to temporarily block the right internal carotid artery to prevent the thrombus from dislodging. Five spring coils (red arrow) were then sequentially placed and released in the aneurysm lumen *via* a microcatheter, and the imaging showed that the spring coils were well formed in the aneurysm lumen. (**D**) After imaging, we still saw extravasation (red arrow) of contrast into the sinus, and continued to use ONYX gel to fill and seal the fistula. (**E**) The imaging shows complete occlusion of the fistula (red arrow) and arterial stent (blue arrow) well released. (**F**) Well reconstruction of the internal carotid artery (red arrow) on imaging and the purpose of embolization is achieved.

A 40-year-old male was diagnosed with pathologically confirmed nasopharyngeal carcinoma (T4N2M0, undifferentiated non-keratinizing carcinoma) (Figure 1). One month later, he was treated with 3D conformal intensity-modulated radiotherapy (63 Gy to the lower neck and 73 Gy to the upper neck) and concurrent chemotherapy (cisplatin 40 mg + docetaxel 120 mg regimen). Three years later, MRI suggested tumor recurrence, and two cycles of GN (gemcitabine + nedaplatin) regimen chemotherapy was performed, after which further heavy proton radiation therapy was administered.Four years later, patient developed an intermittent nasal bleeding. Nine months later, he had a sudden nasal hemorrhage with a bleeding volume of 800 ml, and underwent emergency tracheotomy and bilateral external carotid artery embolization with spring coil. However, hemostasis was not effective. Another emergency DSA was applied, and finally found the real culprit- a pseudoaneurysm of the right internal carotid artery (rupture hole segment (C3)). Then, the operation named “internal carotid artery pseudoaneurysm embolization with stent-assisted spring coil and balloon-assisted onyx gel” was performed. The operation procedure details were shown in Figure 2. Intraoperative angiography showed that the pseudoaneurysm was completely embolized and the internal carotid artery was well reconstructed. The patient was successfully discharged three days after surgery without any neurological deficits. Up to now, the patient did not experience any further nasal bleeding. Time line of the clinical course was shown in Supplementary Figure S1.

### Case two

**Figure 3:**
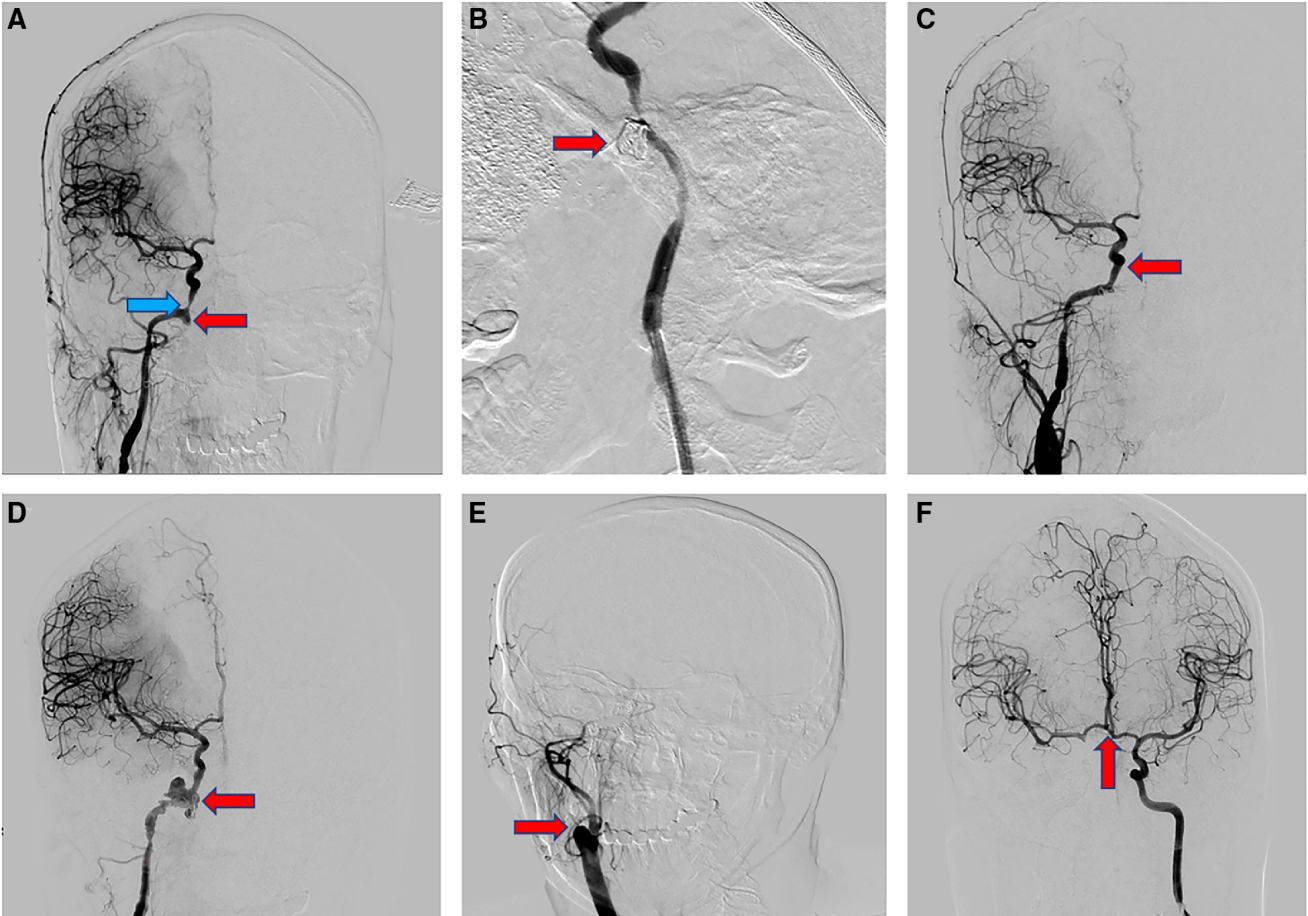
(**A**) the DSA examination showed that the pseudoaneurysm (red arrow) of the internal carotid artery located in the ruptured orifice segment of the right internal carotid artery, with a diameter of about 6*7 mm. The stenosis (blue arrow) of the cavernous sinus segment of the right internal carotid artery was about 50%. (**B**) With the assistance of stent and balloon, the pseudoaneurysm (red arrow) was successfully embolized with spring coils and ONXY gel. (**C**) Well reconstruction of the internal carotid artery (red arrow) on imaging. (**D**) The recurrent pseudoaneurysm (red arrow) located in the C2-4 segment of the right internal carotid artery, with a diameter of about 20*18.8 mm and a lobulated round shape. (**E**) After assessing the compensation of the internal carotid artery on the affected side by compression neck test, we completely and permanently occluded the right internal carotid artery (red arrow) with the aid of a balloon using spring coil and ONYX gel. (**F**) The anterior communicating artery (red arrow) was completely opened on the compression neck test.

A 39-year-old male with pathologically confirmed nasopharyngeal carcinoma (T4N2M0, undifferentiated non-keratinizing carcinoma), and then underwent a combination of chemotherapy and radiotherapy (32 course). The CT examination suggested that the pterygoid body and occipital slope were invaded.One year later, the patient had a sudden onset of massive nasal bleeding with a volume of about 800 ml, and an emergency interventional operation named “internal carotid artery pseudoaneurysm embolization with stent-assisted spring coil and balloon-assisted onyx gel” was performed. The intraoperative angiography showed that the dissection aneurysm on right internal carotid artery C3 segment had been completely embolized, and the aneurysm-carrying artery was still patent.Three months later, however, the patient suffered another nasal hemorrhage with a volume of about 500 ml, and an emergency DSA indicated that the clogged aneurysm was recurrent and larger than before, and located in C2 to C4 segment of the right internal carotid artery. After a hospital-wide multidisciplinary consultation (MDT) discussion, a treatment strategy of permanent occlusion of the aneurysm-carrying artery was decided to effectively prevent the recurrence of nasal hemorrhage. Cerebral angiography and balloon-assisted internal carotid artery occlusion with spring-ring and onyx gel were performed under general anesthesia. Intraoperative angiography showed complete occlusion of the right internal carotid artery, while the right cerebral hemisphere could receive adequate blood supply through the anterior communicating artery. The operation procedure details were shown in Figure 3. Up to now, the patient did not have any further nasal bleeding. Time line of the clinical course was shown in Supplementary Figure S2.

### Discussion

With the improvement of medical technology, the early diagnosis rate and 5-year survival rate of nasopharyngeal carcinoma have increased significantly (1, 10). Nasal hemorrhage caused by ruptured pseudoaneurysm of the internal carotid artery after radiotherapy for nasopharyngeal carcinoma has become one of the most common causes of death after surgery (1, 11). PSA rupture hemorrhage belongs to clinical intensive in otorhinolaryngology and neurosurgery, and related reports claimed that its mortality rate was high (1). The mechanism of its occurrence includes the following aspects: firstly, radiation causes damage to adjacent normal tissues, and the blood vessels invaded by tumor are exposed and become hardened and fibrotic (12); secondly, nasopharyngeal carcinoma can grow to the parapharynx and directly invade the blood vessels in the neck, or indirectly erode the blood vessels by causing nasopharyngeal ulcer and infection, especially recurrent nasopharyngeal carcinoma is mostly located in the pharyngeal fossa, which easily invades the lateral internal carotid artery (11); finally, after the rupture and bleeding of the invaded artery, a hematoma is formed around the rupture of the vessel, which slowly liquefies over time, and the smooth muscle of the middle membrane of the vessel wall becomes thin or even absent, and connects to the aneurysm through the rupture, forming a pulsating pseudotumor (13, 14). Because of the thinness of the wall, it can rupture and bleed, and some patients can stop bleeding on their own when the blood pressure is lowered, thus showing the typical clinical symptoms of repeated bleeding (13).

Due to the concealment of nasopharyngeal bleeding and the special anatomical structure, nasopharyngeal hemorrhage is quite tricky to manage (15). The traditional methods are anterior and posterior nasal tamponade and nasal endoscopic radiofrequency cautery to stop bleeding (16, 17). The anterior and posterior nasal stuffing method is simple to operate and has good efficacy for small amount of nasopharyngeal bleeding, but most patients have limited mouth opening, nasal adhesions, and high risk of rebleeding when the stuffing is removed (17). Radiofrequency cautery under nasal endoscopy is accurate and reliable, but it is difficult to be effective when there is diffuse or rapid bleeding in the nasal cavity (12). In recent years, DSA has gained significant advantages in the treatment of nasopharyngeal carcinoma bleeding, which is the gold standard for identifying the site of vascular bleeding and the presence of vascular malformation (18, 19). Different treatment plans can be implemented according to the importance of the bleeding artery and the bleeding site. When nasopharyngeal carcinoma bleeding is caused by the invasion of the external carotid artery alone, the bleeding artery can be embolized with gelatin sponge particles or spring ring (19); when the tumor invades the internal carotid artery and causes the formation of pseudoaneurysm of the internal carotid artery and rupture bleeding, the pseudoaneurysm can be embolized with stent/balloon assisted spring ring/ONXY glue, or isolation can be performed with overlapping stent; if the pseudoaneurysm is large in scope and difficult to embolize, then unilateral internal carotid artery occlusion is considered (12). But, intraoperative compression angiography of the affected internal carotid artery should be performed to fully assess the opening of the Willis ring, and postoperative attention should be paid to the serious complications (delayed cerebral infarction, etc.) of permanent embolization of the unilateral internal carotid artery (20).

Through the analysis of the cases, we have summarized some treatment experiences: 1) According to the relevant literature, for patients with nasopharyngeal carcinoma who underwent high-dose radiotherapy, multiple radiotherapy and recurrence after radiotherapy, it is especially important to perform regular color ultrasound examination of neck vessels, nasopharyngoscopy and CTA of head and neck for early detection of unruptured PSA (21). After detection, super-selective arterial embolization should be performed as much as possible to reduce embolization of non-diseased arteries. In order to avoid embolic particles from backflowing into the internal carotid artery by mistake and causing serious complications, injection of any kind of embolic material should be done in a low-pressure, slow and intermittent way under x-ray fluoroscopy, and assisted by applying stents or balloons. In the selection of embolic agent, the embolization of PSA in this group of cases is based on spring ring, supplemented by stent if necessary. For larger PSA, in order to achieve more complete embolization and reduce the cost, ONXY gel embolization with the assistance of balloon is applied in this group of cases, which is economical and has good hemostatic effect (22). 2) When patients with nasopharyngeal carcinoma repeatedly bleed heavily within a short period of time after posterior nasal cavity tamponade or interventional embolization of the external carotid artery, they should be alerted to the possibility of PSA rupture and bleeding. The authors suggest that DSA should be performed at an appropriate time to clarify the site of bleeding, and the scope of imaging should include the bilateral internal and external carotid arteries, and to assess the compensation of the Willis loop at the base of the brain (23). During the imaging process, attention should be paid to: whether there is extravasation of contrast agent, which can identify the site of hemorrhage once it appears; whether there is a pseudoaneurysm at the suspected site of hemorrhage; and whether there is vascular stenosis, distortion or disorder at the suspected site of hemorrhage, among which the internal carotid artery mainly shows severe lumen narrowing, suggesting tumor invasion, while the external carotid artery mainly shows vascular distortion and disorder. In addition, in patients with repeated hemorrhage after radiotherapy for nasopharyngeal carcinoma, the bleeding can be temporarily stopped because the blood pressure decreases, the bleeding rate slows down and blood clots are formed. When DSA imaging is performed, the pressure of contrast injection may cause re-bleeding or even violent and fatal hemorrhage, so we advocate to prepare for embolization before DSA, to buy time for resuscitation. 3) When the PSA is not suitable for isolation, it is still controversial whether to perform permanent embolization of unilateral internal carotid artery. Although this method is effective for hemostasis, it may affect the patient's expected survival, cause late cerebral infarction and other complications affecting the patient's quality of life after surgery (24, 25). In our case report, one of the patients accepted permanent unilateral internal carotid artery embolization, meanwhile, the internal carotid artery angiography on the healthy side immediately after embolization indicates that the healthy internal carotid artery has produced compensation, and the patient's quality of life after surgery is good and no large cerebral infarction occurred. However, preforming this method must be chosen carefully after full communication with the patient's family.

## Conclusion

Currently, endovascular intervention is the best option for managing hemorrhage secondary to postoperative internal carotid artery pseudoaneurysm in nasopharyngeal carcinoma patients. However, due to the rapid onset of the disease, emergency DSA is required for diagnosis, and a comprehensive assessment of the internal carotid artery, external carotid artery and intracerebral vascular substitution is also critical. In addition, in order to reduce surgical complications, it is important to detect the responsible vessel as accurately as possible and to select the appropriate embolization protocol and embolization material. Finally, the authors considered that early screening of patients at high risk for PSA and conversion of emergency surgery to elective surgery is the optimal treatment for this disease.

## Data Availability

The original contributions presented in the study are included in the article/**Supplementary Material**, further inquiries can be directed to the corresponding author/s.
