# Supplementary figures and images for: Case report: Endovascular intervention of internal carotid artery pseudoaneurysm secondary to nasopharyngeal carcinoma radiotherapy
Source: Front Surg. 2023 Jan 6;9:1099416. doi: 10.3389/fsurg.2022.1099416 (PMC9879062; doi:10.3389/fsurg.2022.1099416)

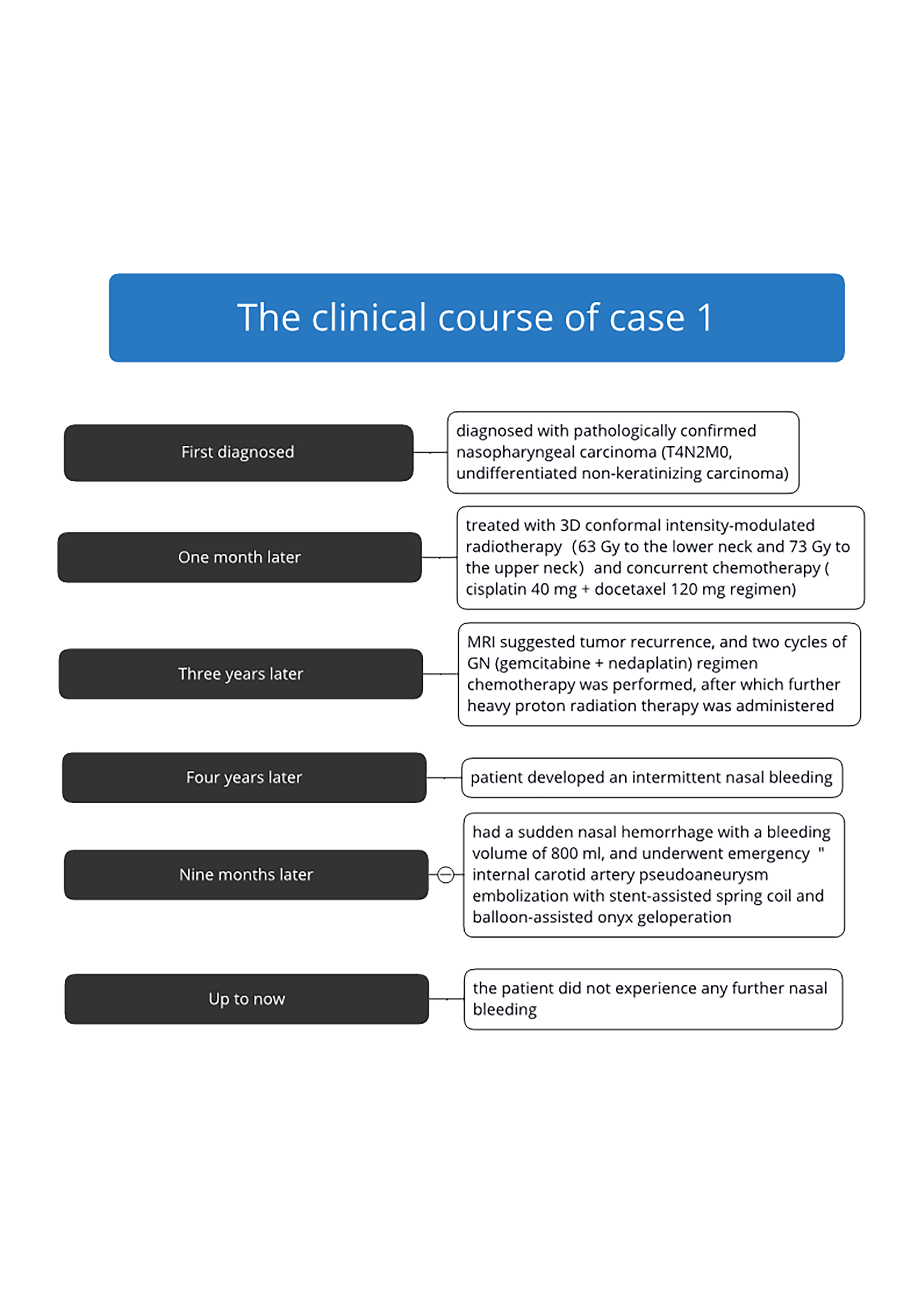

Supplement: Supplementary file 2 [file Image1.tif]

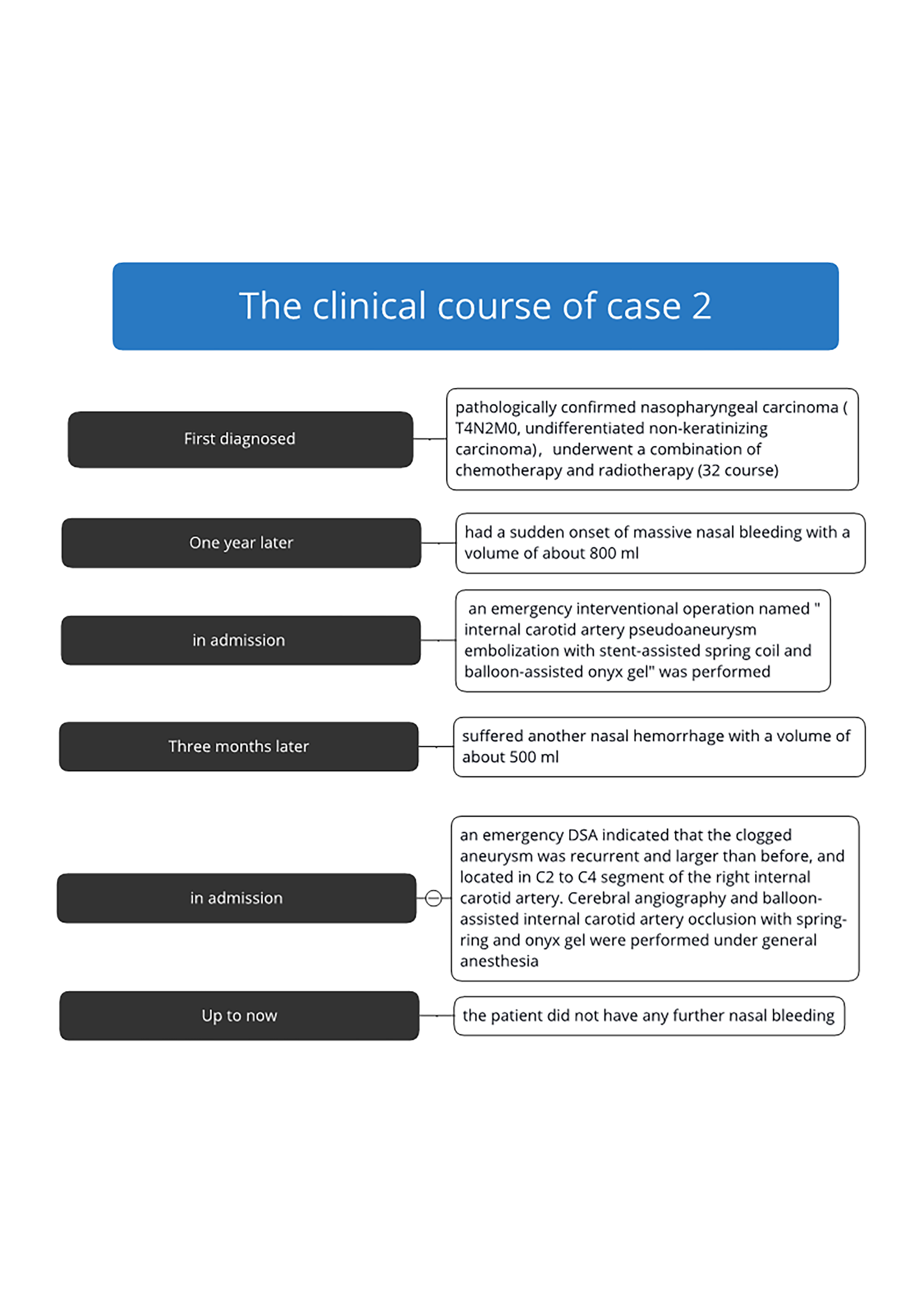

Supplement: Supplementary file 3 [file Image2.tif]
